# Engaging Second Language Learners Using the MUSIC Model of Motivation

**DOI:** 10.3389/fpsyg.2020.01204

**Published:** 2020-06-05

**Authors:** Brett D. Jones

**Affiliations:** Virginia Tech, School of Education, Blacksburg, VA, United States

**Keywords:** L2 instruction, foreign language, second language, motivation, engagement, MUSIC Model of Motivation, motivating students, engagement

## Abstract

The overall aim of this article is to explain how the MUSIC Model of Motivation can be applied to L2 instruction in a manner that is consistent with positive psychology, which emphasizes individuals’ strengths and the conditions in which they thrive. The article begins by describing the MUSIC model, which is a research-based framework that organizes strategies that instructors can use to motivate students to engage in learning. The MUSIC model can be used by L2 instructors to create learning experiences that consider learners’ cognition, affect, needs, and desires in order to foster their motivation and engagement in L2 classes. The article also provides teaching strategies related to the MUSIC model and presents an assessment tool (the MUSIC Model of Academic Motivation Inventory) that can be used by L2 instructors to measure students’ MUSIC perceptions of their class. The assessment tool provides feedback to instructors that can be used to improve their instruction by incorporating strategies that allow their students to flourish. Examples of how the MUSIC Inventory can be used to assess L2 instruction are provided. Although researchers have examined the use of the MUSIC model in L2 classes, this research is underdeveloped, and many questions related to its use in L2 classes remain. The article concludes by proposing unanswered questions that could lead to more effective uses of the MUSIC model in L2 classes.

## Introduction

Learning a second/foreign language (L2) can be interesting and worthwhile; yet, for some students, their experience in L2 courses can be boring and frustrating (Kruk and Zawodniak, 2018). Therefore, some L2 instructors could benefit from understanding principles related to students’ motivation and how to implement motivation strategies in their courses. Researchers have developed several different motivation-related theories over the past few decades that could be useful to instructors (see Dörnyei and Ushioda, 2011; Al-Hoorie, 2017; Al-Hoorie and MacIntyre, 2019; Lamb, 2019). For example, Gardner’s (1985, 2019) Socio-educational Model of Second Language Acquisition identifies several different factors that affect students’ motivation in the learning context and Dörnyei’s (2009) L2 motivational self-system recognizes the importance of the L2 learner’s sense of self and the effects of the L2 learning experience on learners’ motivation (Hadfield and Dörnyei, 2013).

In addition to these types of L2-specific motivation theories, decades of research in the disciplines of psychology, motivation science, and education have led to broader theories of human motivation that have been applied to educational settings (see Schunk et al., 2014; Wentzel and Miele, 2016), including, but not limited to, self-efficacy theory (Bandura, 1986), arousal theories (Berlyne, 1960), flow theory (Csikszentmihalyi, 1990), self-determination theory (Deci and Ryan, 1985), self-theories of intelligence (Dweck, 1999), interest theories (Hidi and Renninger, 2006), control-value theory (Pekrun, 2009), attribution theory (Weiner, 2000), and expectancy-value theory (Wigfield and Eccles, 2000). Researchers have applied these theories in L2 settings (e.g., self-determination theory, Jones et al., 2009; Noels et al., 2019), which demonstrates the utility of applying broader motivation theories to L2 education. Researchers have also begun to study the role of positive psychology—the study of the positive aspects of human life—in L2 education (e.g., flow theory, Gregersen, 2019; Piniel and Albert, 2019).

The overall aim of this article is to discuss how a conceptual model of motivation can be applied to L2 instruction in a manner that is consistent with positive psychology, which emphasizes individuals’ strengths and the conditions in which they thrive. To achieve this goal, I describe the MUSIC Model of Motivation (Jones, 2009, 2018) and how it can be used by L2 instructors to create learning experiences with a consideration of learners’ cognition, affect, needs, and desires in order to foster their motivation and engagement in L2 classes. The MUSIC model does not replace existing L2 motivation theories, but rather, it can be used as a complementary approach that focuses on what Lamb (2019) referred to as “*motivating* learners, as opposed to learner *motivation*” (p. 288).

## The Music Model of Motivation

The MUSIC^®^ Model of Motivation (Jones, 2009, 2018) provides instructors with a research-based framework that summarizes effective motivational strategies for instructors. The key principles of the MUSIC model are that “the instructor needs to ensure that students:

1. Feel empowered by having the ability to make decisions about some aspects of their learning,
2. Understand why what they are learning is useful for their short- or long-term goals,
3. Believe that they can succeed if they put forth the effort required,
4. Are interested in the content and instructional activities, and
5. Believe that others in the learning environment, such as the instructor and other students, care about their learning and about them as a person” (Jones, 2018, p. 9).

The acronym MUSIC is used to help instructors remember the initial sounds of these five groups of strategies: eMpowerment, Usefulness, Success, Interest, and Caring.

**FIGURE 1:**
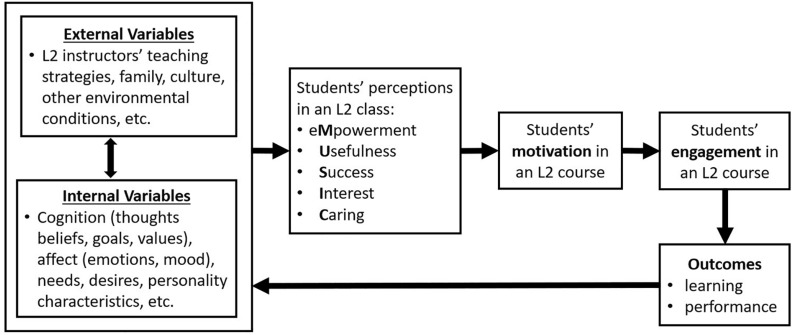
This figure shows a simplified version of how external and internal factors affect L2 students’ perceptions of the MUSIC Model of Motivation components, which then affect their motivation, engagement, and learning/performance, which then cycles back and affects the external and internal factors. Adapted from “Motivating Students by Design: Practical Strategies for Professors” by B. D. Jones, 2018, p. 13. Copyright 2018 by Brett D. Jones. Reprinted with permission.

In the MUSIC model, motivation is defined as “the extent to which one intends to engage in an activity” (Jones, 2018, p. 5), and thus, it precedes engagement. Engagement is defined as participating in an activity, either behaviorally (e.g., taking notes in a class) or cognitively (e.g., thinking about class ideas) (Jones, 2018, p. 6). Students’ motivation and engagement are affected by their perceptions of the class, which are influenced by external variables (i.e., variables that are outside students’ bodies, such as teaching strategies, family, culture, etc.) and internal variables (see Figure 1). The goal of the instructor is to use teaching strategies that create conditions in the class to motivate students to engage in the learning activities. Through positive engagement in class over time, students are more likely to learn and perform at higher levels and to develop more productive cognition and affect related to the L2 and learning activities (e.g., “I can learn to speak Spanish,” “I like learning Spanish”). As a result, students are more likely to meet the course objectives and have a positive experience while doing so. Figure 1 shows how these variables form a cycle in which the instructor participates by implementing teaching strategies that motivate and engage students in ways that help the students flourish over time. This social, contextual, and dynamic approach to motivation is consistent with approaches advocated by some L2 researchers (e.g., Dörnyei and Ushioda, 2011; Dörnyei et al., 2015; Yim et al., 2019).

### Teaching Strategies Related to the MUSIC Model

In the MUSIC model, the key to motivating students in classes is to positively affect their MUSIC perceptions (i.e., perceptions of empowerment, usefulness, success, interest, and caring). Empowerment strategies include those that give students choices and control in the class. For example, L2 teachers can give students choices among or within assignments and incorporate student-centered approaches that provide students with some autonomy (Jones, 2009, 2016). Usefulness strategies help students to understand the usefulness and benefits of what they are learning, either in the short- or long-term. For instance, L2 teachers can use real-life and practical examples and ask students or others (former students, professionals) to share the reasons they find the course content useful. Success strategies lead students to believe that they can succeed if they put forth effort. Examples include L2 teachers (a) providing regular, specific feedback that can help students to improve; (b) matching the difficulty level of the class assignments with the abilities of the students; and (c) attributing students’ struggles to their lack of effort or to their use of ineffective study strategies and then helping them to use more effective strategies (Jones, 2018). Interest strategies are used to engage students cognitively and affectively by creating enjoyable and interesting activities. Teachers can accomplish this by designing activities that grab students’ attention, pique their curiosity about the content, and arouse them emotionally. Caring strategies ensure that students believe that their teacher cares about their success and that they are treated with respect by their teacher and classmates. Teachers can foster perceptions of caring by being approachable and relatable to students, as well as by ensuring that students in the class treat each other fairly and with respect.

Although the MUSIC model was designed to be applied by instructors in any discipline, the model is consistent with motivational teaching strategies proposed by L2 researchers. For example, Dörnyei and Ushioda (2011) note that it is important for L2 teachers to create learner autonomy and promote self-motivation (the eMpowerment component in the MUSIC model) and to help students understand the relevance of activities and set goals so they can better understand why what they are learning is important (the Usefulness component). They also recommend increasing learners’ expectancy of success and self-confidence as learners (the Success component), getting students curious about and interested in course activities and in foreign languages more generally (the Interest component), and fostering teacher-student relationships and promoting group cohesiveness (the Caring component).

### Evidence for the MUSIC Model

Many studies have been conducted to demonstrate that (1) students’ MUSIC perceptions of a class (i.e., perceptions of empowerment, usefulness, success, interest, and caring) are related, but distinct (Jones and Wilkins, 2013; Jones and Skaggs, 2016b; Chittum and Jones, 2017); (2) students’ MUSIC perceptions are related to their engagement in the class (Jones, 2010, 2019; Jones and Carter, 2019); and (3) teachers and researchers can assess students’ MUSIC perceptions in a class and for specific learning activities (Chittum et al., 2017; Jones and Cruz, 2017). The research supporting these findings is quite robust given that studies have been conducted across disciplines and age levels.

### Assessment of the MUSIC Model Components

Instructors can assess their motivational strengths and weaknesses in a class in a variety of ways, such as by surveying students, reflecting on their teaching experiences, talking to students, and soliciting feedback from colleagues about their instruction (Jones, 2018). In this section, I focus on assessment using surveys by describing quantitative and qualitative questionnaire items that have been used by teachers and researchers.

A questionnaire, titled the MUSIC^®^ Model of Academic Motivation Inventory (Jones, 2012/2020), is available for free^1^ and provides instructors with scores ranging from 1 to 6 on each of the MUSIC model components: eMpowerment, Usefulness, Success, situational Interest, and Caring. The MUSIC Inventory has been shown to produce valid scores with students when considering Cronbach’s alpha values (α) and fit indices from factor analyses in samples from elementary school (Jones and Sigmon, 2016a, M α = 0.72, U α = 0.71, S α = 0.65, I α = 0.76, and C α = 0.64), middle and high school (Parkes et al., 2017, M α = 0.73, U α = 0.86, S α = 0.92, I α = 0.91, and C α = 0.92), college (Jones and Skaggs, 2016b, M α = 0.91, U α = 0.96, S α = 0.93, I α = 0.95, and C α = 0.93), and professional schools (Jones et al., 2019, M α = 0.85, U α = 0.88, S α = 0.88, I α = 0.80, and C α = 0.82 for sample 2; Pace et al., 2016, M α = 0.89, U α = 91., S α = 0.92, I α = 0.91, and C α = 0.92) (see Jones, 2012/2020 for more complete validity evidence). The MUSIC Inventory has been translated to a variety of languages and preliminary evidence indicates that it produces valid scores when used in L2 courses in China, Egypt, Iran, and Mexico (Jones et al., under review). Qualitative items have also been used to ask students about their MUSIC perceptions (see Jones, 2012/2020). An example item is: What could be changed in this course to make it more interesting and enjoyable?

**FIGURE 2:**
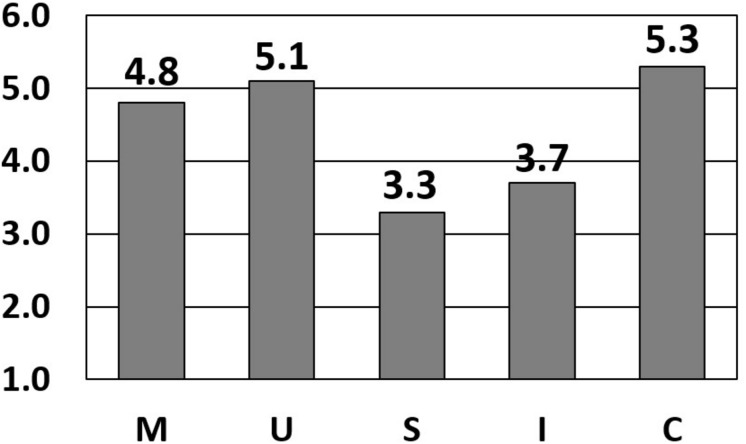
Example scores obtained from using the MUSIC Inventory within a course.

An example of the five MUSIC scale scores produced from the MUSIC Inventory is shown in Figure 2 and instructors can use these scores to identify their motivational strengths and weaknesses. For example, teachers whose students rate them low on success and interest (as shown in Figure 2) could consider incorporating strategies that increase students’ perceptions of success and interest in the class.

One way to use the MUSIC Inventory is to collect data near the end of a course and then use the results to improve the course in the future. Le et al. (2014) used the MUSIC Inventory in this way to assess the MUSIC perceptions of 197 students in a Medical English course (an English for Specific Purposes course) at a medical university in Vietnam. Some students also participated in focus group interviews after they completed the inventory. Findings indicated that students were generally motivated in the course, but that some aspects of the course could be improved. For instance, student feedback suggested that instructors could empower students more by providing them with more choices related to how to achieve the goals of the course. Overall, they concluded that the “MUSIC Model is also believed to be a practically helpful idea for those delivering ESP courses in non-English major universities” (p. 724).

In another study, Li and her colleagues (Li et al., 2016) used the MUSIC Inventory near the end of a college English class in China to examine the relationships between students’ MUSIC perceptions and their engagement. They found that students reported lower scores on the empowerment, success, and interest scales and that all of the MUSIC scales were significantly correlated with students’ engagement. They concluded that “it is possible for Chinese college EFL teachers to use the strategies in the MUSIC model to redesign their instruction to motivate and engage their students in the English coursework” (p. 1767).

Another way to use the MUSIC Inventory is to collect data at multiple time points throughout a course. Doing so allows the instructor or researcher to track how students’ MUSIC perceptions change over time. It also allows instructors to alter their teaching strategies during a course or to develop an intervention to address weaknesses that may arise before the course is completed. As an example, Li and Jones (2019) implemented an intervention during a course based on the results of the MUSIC Inventory that were obtained early in the course. Three instructors administered the MUSIC Inventory during the second week of one of their L2 classes and students reported lower perceptions of empowerment, success, and interest. To increase these student perceptions, they developed an intervention that used student group presentations and an associated rubric to grade the presentations. After this 10-week intervention, students completed the MUSIC Inventory again and students’ ratings on all five MUSIC model components were statistically higher after the intervention than before the intervention.

Furthermore, experimental studies with control groups have been conducted in other disciplines to demonstrate that interventions that focus on one or more MUSIC-related constructs can have a positive effect on students’ MUSIC perceptions and engagement (Reeve et al., 2004; McGinley and Jones, 2014; Lin-Siegler et al., 2016). For example, when students in an experimental group were asked to write essays about how the class material was useful to their lives, students with lower expectations were more likely than a control group to increase their success expectations, become more interested in the content, and achieve higher in that class (Hulleman et al., 2017).

## Discussion

### Designing Instruction Using the MUSIC Model

One purpose of this article was to present the MUSIC model as a framework that can be used by L2 instructors to design learning experiences that motivate and engage students. Consistent with the tenets of positive psychology, the MUSIC model can be used to create conditions in which students can thrive by supporting their cognition (thoughts, beliefs, goals, values), affect (emotions, moods), needs, and desires. Conceptually, the MUSIC model is a promising means to engage students in L2 classes because it is based on well-established motivation theories. Although the MUSIC model has been used in a variety of disciplines and grade levels, many questions remain about how the MUSIC model can be most effectively used in L2 classes, including:

1. Are some MUSIC model components more important than others to engage students? In examining the MUSIC model components in eight different undergraduate courses, Jones (2019) found that the number of MUSIC components that were significantly correlated to effort ranged from two components to all five components, depending upon the course.
2. Is it more difficult to implement some components of the MUSIC model in some cultures than others? For instance, it may be more difficult to give students empowerment in Eastern cultures if they are more likely than Western cultures to rely on traditional lecture approaches (with relatively no empowerment).
3. Do increases in one or more of the MUSIC model components lead to increases in other components? In one study (Hulleman et al., 2017), increasing students’ perceptions of usefulness also increased their perceptions of success and interest.

Understanding the answers to these types of questions could help instructors more effectively plan their instruction to target the most important MUSIC strategies.

### Assessing Instruction Using the MUSIC Inventory

Another purpose of this article was to describe ways in which the MUSIC Inventory can be used by L2 instructors to measure students’ MUSIC perceptions in class. The MUSIC Inventory and other open-ended questions related to the MUSIC model components have been successfully used by L2 instructors to provide data that can be used to redesign their instruction. However, questions as to how the inventory can be used most effectively in L2 classes remain, such as:

1. Is it important to collect both quantitative MUSIC Inventory data and qualitative open-ended item data or is it sufficient to collect just one of these? Some researchers have administered open-ended items for each of the MUSIC components in order to obtain feedback that can be used to improve instruction (Jones et al., 2012).
2. Can other data be used to complement the MUSIC data? Other possibilities that could help instructors understand students’ motivation in a course include students’ self-reported effort, cognitive engagement, and identification with a domain (Jones et al., 2016; Jones and Carter, 2019).
3. When is the most effective time within a course to collect MUSIC data? Collecting data in the middle of a course can be useful in predicting students’ overall course effort, instructor rating, and course rating at the end of the course (Jones, 2019) and may be used by the instructor to improve the course. However, by also collecting data at the end of the course, instructors can assess *changes* in students’ perceptions during the course, especially if data is collected at multiple time points throughout a course.

Answering these types of questions could be helpful to both instructors and researchers as they strive to collect useful data.

## Conclusion

Using strategies related to each of the MUSIC model components appears to be a promising means to create a class environment that promotes student engagement and allows students to flourish. The MUSIC model provides an organizational framework that teachers can use to design and evaluate their instruction. However, many questions remain about how the MUSIC model can be used most effectively by L2 teachers within different contexts. Through rigorous empirical study, researchers and instructors can answer many of these questions, which will allow instructors to create L2 classes and activities that promote positive student growth and learning.

## Author Contributions

BJ is the sole author of this article and is accountable for the content of this article.

## Conflict of Interest

The author declares that the research was conducted in the absence of any commercial or financial relationships that could be construed as a potential conflict of interest.

## Footnotes

1 www.theMUSICmodel.com
